# Chronic, Environmentally Relevant PM_2.5_ Exposure Exacerbates Metabolic Dysfunction‐Associated Steatotic Liver Disease and Early‐Stage Renal Dysfunction in a Rodent Model

**DOI:** 10.1002/kjm2.70206

**Published:** 2026-03-28

**Authors:** Yi‐Siao Chen, Hugo You‐Hsien Lin, Chien‐Tzu Lin, Si‐Hua Huang, Ming‐Lun Yeh, Ming‐Lung Yu, Jin‐Ching Lee, Yao‐Chi Chung, Wei‐Ming Li, Ching‐Chia Li, Shiu‐Feng Huang, Chia‐Hung Yen

**Affiliations:** ^1^ Ph.D. Program in Environmental and Occupational Medicine, College of Medicine Kaohsiung Medical University and National Health Research Institutes Kaohsiung Taiwan; ^2^ Institute of Molecular and Genomic Medicine National Health Research Institute Miaoli County Taiwan; ^3^ Graduate Institute of Natural Products, College of Pharmacy Kaohsiung Medical University Kaohsiung Taiwan; ^4^ Drug Development and Value Creation Research Center Kaohsiung Medical University Kaohsiung Taiwan; ^5^ Division of Nephrology, Department of Internal Medicine Kaohsiung Medical University Hospital Kaohsiung Taiwan; ^6^ Department of Medicine, College of Medicine Kaohsiung Medical University Kaohsiung Taiwan; ^7^ Center of Excellence for Metabolic Associated Fatty Liver Disease National Sun Yat‐sen University Kaohsiung Taiwan; ^8^ Hepatobiliary Division, Department of Internal Medicine Kaohsiung Medical University Hospital Kaohsiung Taiwan; ^9^ Center of Hepatitis Research, College of Medicine and Center of Metabolic Disorders and Obesity Kaohsiung Medical University Kaohsiung Taiwan; ^10^ Department of Marine Biotechnology and Resources National Sun Yat‐sen University Kaohsiung Taiwan; ^11^ Department of Biotechnology, College of Life Science Kaohsiung Medical University Kaohsiung Taiwan; ^12^ Center for Tropical Medicine and Infectious Disease Research Kaohsiung Medical University Kaohsiung Taiwan; ^13^ Graduate Institute of Animal Vaccine Technology, College of Veterinary Medicine National Pingtung University of Science and Technology Pingtung Taiwan; ^14^ Department of Urology Kaohsiung Medical University Gangshan Hospital Kaohsiung Taiwan; ^15^ Department of Urology Kaohsiung Medical University Hospital, Kaohsiung Medical University Kaohsiung Taiwan; ^16^ Department of Urology, School of Medicine, College of Medicine Kaohsiung Medical University Kaohsiung Taiwan

**Keywords:** animal model, chronic kidney disease, metabolic dysfunction‐associated steatotic liver disease, metabolic syndrome, PM_2.5_

## Abstract

While epidemiological studies link fine particulate matter (PM_2.5_) exposure to metabolic dysfunction‐associated steatotic liver disease (MASLD) and renal dysfunction, a translational gap exists, as most animal models utilize acute, high‐dose exposures that poorly reflect chronic, moderate‐level human scenarios. To address this, we established a long‐term (seven‐month) mouse model (*n* = 8 per group) combining a Western diet (WD) with a chronic PM_2.5_ exposure paradigm equivalent to a human exposure of ~50 μg/m^3^, assessing key biomarkers, organ histopathology, and hepatic gene expression. While the WD was the primary driver of MASLD‐related steatosis and insulin resistance, PM_2.5_ co‐exposure acted as a distinct modifier of hepatic pathogenesis. Specifically, the WD + PM group exhibited significantly exacerbated hepatic pathology compared to WD alone, characterized by a ~55% increase in serum ALT (*p* < 0.05) and a 4.7‐fold increase in collagen deposition area (*p* < 0.05), alongside the marked upregulation of pro‐inflammatory (TNF‐α, CCL2) and pro‐fibrotic (α‐SMA, COL1A1) genes. In the kidney, PM_2.5_ exposure independently elevated serum creatinine levels (by ~64% vs. controls, *p* < 0.05) and increased the incidence of proteinuria (75% in WD + PM vs. 0% in controls). These functional alterations occurred without inducing major structural damage. This study provides crucial experimental evidence that moderate, chronic air pollution aggravates diet‐induced hepatic inflammation/fibrosis and contributes to early‐stage renal dysfunction, underscoring the multi‐organ health threats to metabolically vulnerable populations.

## 1 Introduction

Metabolic dysfunction‐associated steatotic liver disease (MASLD) has become a pressing global health issue, with a prevalence affecting approximately 30% of the world's population [1]. Beyond its high prevalence, MASLD is a progressive condition that can lead to severe liver‐related complications, including cirrhosis, hepatocellular carcinoma, and the eventual need for liver transplantation, positioning it as a major contributor to chronic liver disease burden worldwide [2]. The pathogenesis of MASLD is complex and multifactorial, driven by genetic predisposition, dietary habits, and metabolic dysfunction [3, 4]. Recently, environmental factors, particularly air pollution, have emerged as significant and modifiable risk factors contributing to the onset and progression of MASLD [5, 6]. Moreover, air pollution can complicate the clinical management of chronic liver diseases by promoting progression to hepatocellular carcinoma (HCC) [7]. Among environmental pollutants, fine particulate matter (PM_2.5_) is a primary concern.

A growing body of epidemiological evidence has established PM_2.5_'s role as a key environmental risk factor for metabolic syndrome, MASLD, and even chronic kidney disease (CKD) [5, 8, 9, 10]. Specifically, recent meta‐analyses and large‐scale studies have shown that each 10 μg/m^3^ increase in long‐term PM_2.5_ exposure is associated with an approximately 8% increased risk for metabolic syndrome [9], a 29% increased risk for MASLD [6], and a 31% increased risk for incident CKD [10]. Crucially, these associations were documented in populations with median annual mean PM_2.5_ concentrations of 25, 41.1, and 63.7 μg/m^3^ (see references [7, 8, 10], respectively). To place these real‐world concentrations into a clinical context understandable to the general public, we referenced the U.S. EPA's Air Quality Index (AQI) breakpoints. According to the Technical Assistance Document for the Reporting of Daily Air Quality [11], these concentrations correspond to AQI categories ranging from ‘Moderate’ (AQI 51–100; 9.1–35.4 μg/m^3^) to ‘Unhealthy’ (AQI 151–200; 55.5–125.4 μg/m^3^). While we acknowledge the distinction between annual means used in epidemiology and daily reporting metrics, this mapping illustrates that metabolic risks are prevalent even under moderate pollution episodes, contrasting with the extreme toxicological events often utilized in previous animal studies (e.g., 115–375 μg/m^3^ or 10 mg/kg; typically exceeding AQI 200) [12, 13, 14, 15, 16].

MASLD's impact extends to the kidney, with CKD as a frequent complication [17, 18, 19]. While prior animal studies have reported that high‐concentration PM_2.5_ exposure (e.g., 135.90 μg/m^3^ or 5 mg/kg) can synergize with a high‐fat diet to exacerbate renal injury [20, 21], it remains unclear whether chronic exposure to “moderate” levels of PM_2.5_—which may not be overtly toxic on their own—can induce similar synergistic susceptibility. MASLD is closely linked to CKD [18, 19], and PM_2.5_ is an independent risk factor for both [5, 10]. Therefore, we hypothesized that diet‐induced metabolic dysfunction acts as an effect modifier, increasing physiological susceptibility to environmental pollutants. To address this knowledge gap, we employed a long‐term mouse model combining a Western diet with chronic, environmentally relevant PM_2.5_ exposure (~50 μg/m^3^) to evaluate the interaction between these stressors, specifically determining whether air pollution exacerbates hepatic and renal injury under conditions of metabolic stress.

## 2 Materials and Methods

### 2.1 Animal Experiments

Six‐week‐old male C57BL/6J mice (weighing approximately 20 ± 2 g) were obtained from the National Laboratory Animal Center, Taiwan. All animal experiments were conducted in accordance with the Animal Research: Reporting of In Vivo Experiments (ARRIVE) guidelines. The study protocol was reviewed and approved by the Institutional Animal Care and Use Committee (IACUC) of Kaohsiung Medical University (IACUC #111069), and all animal procedures were performed in accordance with the Guide for the Care and Use of Laboratory Animals published by the National Research Council (NIH). The mice were housed in the animal care facility at Kaohsiung Medical University under controlled conditions: a temperature of 21°C ± 2°C, relative humidity of 55%–70%, and a 12‐h light/dark cycle. After a one‐week adaptation period, the animals were randomly assigned to one of four groups (*n* = 8 per group) using a computer‐generated randomization sequence and treated according to the following protocols for 7 months: normal diet (ND), Western diet (WD), ND with PM_2.5_ exposure (ND + PM), and WD with PM_2.5_ exposure (WD + PM).

In this study, mice in the ND and ND + PM groups were fed a normal rodent diet (D12450B; 3.85 kcal/g, with 10% of calories from fat, 20% from protein, and 70% from carbohydrates). Meanwhile, mice in the WD and WD + PM groups were fed a Western diet (Inotiv Inc., TD.120528, West Lafayette, Indiana, USA), which consisted of 21.2% fat, 40% sucrose, and 1.25% cholesterol by weight. Additionally, mice in the WD and WD + PM groups received sugar water (23.1 g/L D‐fructose and 18.9 g/L D‐glucose) as drinking water, while mice in the ND and ND + PM groups were provided with regular water. PM_2.5_ (Standard Reference Material 1650b, Sigma‐Aldrich, St. Louis, MO, USA) was used for exposure. This material is a well‐characterized Diesel Particulate Matter provided by the National Institute of Standards and Technology (NIST). According to its certificate of analysis, it is sourced from the exhaust of heavy‐duty diesel engines and serves as a reference standard for the analysis of polycyclic aromatic hydrocarbons (PAHs) and other pollutants. Critically for this study, its particle size distribution has a volume median diameter of approximately 0.18 μm after sonication, confirming it consists predominantly of fine and ultrafine particles capable of deep respiratory penetration. The PM_2.5_ was dissolved in 0.01% Tween 80 in PBS and administered to the mice via tracheal instillation (three times per week, 0.5 mg/kg). Mice in the ND and WD groups were also administered 0.01% Tween 80 in PBS via tracheal instillation as a control. Body weight was recorded weekly for each group. The rationale for the PM_2.5_ dosage was derived using body surface area (BSA) normalization to approximate human exposure levels, following FDA guidelines. The calculation workflow is as follows: Mice received 0.5 mg/kg via intratracheal instillation three times per week, corresponding to an average daily dose of approximately 0.21 mg/kg/day. To convert this to a human equivalent dose (HED), we applied the standard species conversion factor of 12.3 (mouse Km = 3, human Km = 37), resulting in an HED of approximately 0.017 mg/kg/day (0.21/12.3). For a standard 60 kg adult, this translates to a total daily intake of 1.02 mg (1020 μg). Assuming a daily ventilation volume of 20 m^3^ for an adult engaged in moderate physical activity, this daily burden corresponds to an ambient PM_2.5_ concentration of approximately 51 μg/m^3^ (1020 μg/20 m^3^). Thus, our experimental dosage effectively models the cumulative systemic burden corresponding to chronic human exposure at a daily average concentration of ~50 μg/m^3^. Furthermore, considering the accelerated life history of mice relative to humans, the 7‐month exposure period covering the transition from young adulthood to middle age is broadly equivalent to approximately 20 human years. This estimate is based on established comparative aging models [22], which posit that mice mature approximately 45 times faster than humans during early adulthood (up to 6 months) and age 25 times faster thereafter. Thus, our model reflects a significant portion of the lifespan, mimicking a chronic, long‐term exposure scenario.

### 2.2 Intraperitoneal Glucose Tolerance Testing (IPGTT)

To test glucose tolerance, mice were fasted for 12 h before receiving an intraperitoneal administration of 2 g/kg glucose load. Their blood glucose was measured before (0 min) and 15, 30, 60, and 120 min after glucose load administration, from the tip of their tails using a blood glucose meter (Accu‐chek Instant, Roche, Basel, Switzerland). The area under the curve (AUC) for glucose levels during the IPGTT was calculated using GraphPad Prism software (version 8.0).

### 2.3 Urine Collection and Handling

One week prior to sacrifice, mice were individually housed in metabolic cages to collect urine samples for renal function assessment. To minimize stress‐induced physiological variations, mice were subjected to a 24‐h acclimatization period immediately followed by a 24‐h sample collection period. Throughout this entire 48‐h duration, mice were provided with ad libitum access to their respective experimental diets (ND or WD) and water to ensure a stable metabolic state consistent with their long‐term feeding regimen. Urine was collected in sterile tubes at ambient temperature. To ensure sample integrity, samples were retrieved immediately after the 24‐h period and centrifuged at 3000 rpm for 10 min at 4°C to remove particulate matter and debris. The supernatant was then immediately aliquoted: fresh supernatant was used for dipstick analysis, while the remainder was stored at −80°C for subsequent biochemical analyses (uACR, KIM‐1). Urinalysis was performed using QTEST 11 reagent strips (Madimpex United Inc., Bensalem, PA, USA) by a physician who was blinded to the experimental grouping code. The detection is based on the “protein error of indicators” principle, specifically targeting albumin. A visual color change from yellow (negative) to yellow‐green or blue‐green was interpreted according to the manufacturer's color chart. Based on the manufacturer's specifications that “Trace” levels or higher indicate significance, proteinuria was defined as ‘positive’ for readings corresponding to ≥ 15 mg/dL (Trace to 4+). Due to the ad libitum feeding protocol, strict quality control was applied; samples with visible food/fecal contamination were excluded from the analysis.

### 2.4 Serum and Urine Analysis

During animal sacrifice, blood was collected and allowed to coagulate for 30 min. Following blood coagulation, serum was collected after centrifugation at 2300 rcf for 15 min and stored in a −80°C freezer. Levels of creatinine, alanine aminotransferase (ALT), total cholesterol (TCHO), triglycerides (TG), and high‐density lipoprotein cholesterol (HDL‐C) were analyzed on an automatic dry‐chemistry analyzer (Dri‐Chem NX‐500, Fujifilm Corporation, Tokyo, Japan) according to manufacturer instructions. Serum insulin levels were measured by ELISA (Ultra‐Sensitive Mouse Insulin ELISA Kit, #90080, Crystal Chem, Itasca, IL, USA). The Homeostatic Model Assessment of Insulin Resistance (HOMA‐IR) index was calculated using the following formula: HOMA‐IR = [Fasting Blood Glucose (mg/dL) × Fasting Serum Insulin (mIU/L)]/405. Urine was collected using a metabolic cage, and levels of kidney injury molecule‐1 (KIM‐1), creatinine, and microalbuminuria were measured by ELISA (KIM‐1 ELISA Kit, ab213477, abcam, Cambridge, UK; Creatinine ELISA Kit, ELK9151, ELK Biotechnology, Wuhan, Hubei, China; Microalbuminuria ELISA Kit, ELK9430, ELK Biotechnology, Wuhan, Hubei, China).

### 2.5 Quantitative Real‐Time PCR (QPCR)

RNA was prepared by using TOOLSmart RNA Extractor (TOOLs Biotechnology, New Taipei City, Taiwan) and was reverse transcribed into cDNA using a High‐Capacity cDNA Reverse Transcription Kit (Thermo Fisher Scientific, Sunnyvale, CA, USA). QPCR was performed on an ABI StepOne Plus System (Applied Biosystems, Foster City, CA, USA) using the TOOLS 2X SYBR qPCR Mix (TOOLs Biotechnology, New Taipei City, Taiwan). The mRNA level was normalized to the TATA‐box binding protein (TBP) mRNA level. The following primers were used: TGF‐β (NM_011577.2) forward: 5′‐ATACGCCTGAGTGGCTGTCT and reverse: 5′‐TCATGGATGGTGCCCAGGTC; α‐SMA (NM_007392.3) forward: 5′‐ACTGGGACGACATGGAAAAG and reverse: 5′‐GTTCAGTGGTGCCTCTGTCA; COL1A1 (NM_007742.4) forward: 5′‐TGACTGGAAGAGCGGAGAGTA and reverse: 5′‐AGACGGCTGAGTAGGGAACA; CCL2 (NM_011333.3) forward: 5′‐TTTTGTCACCAAGCTCAAGAGAG and reverse: 5′‐TCACTGTCACACTGGTCACTCC; TNF‐α (NM_013693.3) forward: 5′‐GTCCCCAAAGGGATGAGAAGT and reverse: 5′‐TTTGCTACGACGTGGGCTAC; IL‐1β (NM_008361.4) forward: 5′‐GAAATGCCACCTTTTGACAGTG and reverse: 5′‐TGGATGCTCTCATCAGGACAG; MMP13 (NM_008607.2) forward: 5′‐AGAAGTGTGACCCAGCCCTA and reverse: 5′‐GCGCCAGAAGAATCTGTCTTT; TIMP1 (NM_001044384.2) forward: 5′‐CAGAAATCAACGAGACCACCTT and reverse: 5′‐GCCTTGAATCCTTTTAGCATCTTA; TBP (NM_013684.3) forward: 5′‐GCACAGGAGCCAAGAGTGAAGA and reverse: 5′‐TCCCCACCATGTTCTGGATCT.

### 2.6 Histopathology

For the histopathological examinations, hematoxylin and eosin (H&E) and Masson's trichrome staining were performed. Briefly, liver and kidney tissues were fixed in 10% neutral buffered formalin, processed for standard paraffin embedding, sectioned (5 μm), and deparaffinized for H&E or Masson's trichrome staining (TASS01‐250, BioTnA) following the manufacturer's instructions. These sections were stained with H&E and mounted with DPX for histopathological analysis. For Masson's trichrome staining, the procedure was carried out using a commercial kit (TASS01‐250, BioTnA; Kaohsiung, Taiwan) following the manufacturer's instructions.

### 2.7 Histological Quantification and Image Analysis

Quantitative image analysis was performed using the Fiji distribution of ImageJ software (version 1.54p; National Institutes of Health, USA). To ensure methodological rigor and minimize bias, image acquisition and subsequent analyses were performed in a blinded manner, with the investigator unaware of the experimental grouping. Images were acquired from 8 mice per group. For each tissue section, 5 randomly selected non‐overlapping fields of view were captured using a 20× objective (200× magnification). To quantify the severity of hepatic steatosis, H&E‐stained sections were analyzed to measure the area of lipid vacuoles. The green channel was extracted from the RGB images to maximize the contrast between lipid droplets and the surrounding parenchyma. An adaptive threshold was applied to segment high‐intensity areas corresponding to lipid vacuoles. The watershed algorithm was employed to separate clustered droplets. To ensure specificity, a circularity filter (0.60–1.00) was strictly applied to distinguish spherical lipid droplets from vascular structures and sinusoidal spaces. Particles smaller than 10 pixels were excluded to minimize noise. The degree of steatosis was expressed as the percentage of lipid droplet area relative to the total field area (Steatosis area %). To assess hepatic inflammation, immune cell infiltration was quantified based on nuclear staining. Images were processed using the Color Deconvolution plugin [23] with standard vectors. The hematoxylin channel (Colour_1), representing nuclear signals, was selected for analysis. To enhance feature separation and reduce background noise, morphological filtering was performed using the MorphoLibJ plugin [24]; specifically, a morphological opening operation was applied using a disk structuring element with a radius of five pixels. A manual threshold was applied to each image to define positive nuclear areas and adjusted to account for slight variations in staining intensity. Binary masks were generated, and the extent of inflammation was calculated as the percentage of the area occupied by inflammatory nuclei (Inflammatory infiltration %). Masson's trichrome‐stained sections were analyzed using the Color Deconvolution plugin. The blue channel, corresponding to collagen fibers, was isolated. A threshold was applied to segment the positive collagen areas (Collagen area). Simultaneously, the total tissue area was determined by thresholding the original image to exclude the background (Total tissue area). The severity of fibrosis was calculated as the percentage of the collagen‐positive area relative to the total tissue area (Fibrosis %). All quantitative data were averaged for each animal prior to statistical analysis.

### 2.8 Statistical Analysis

Data are presented as mean ± SEM (standard error of the mean). Statistical analyses were performed using GraphPad Prism software version 8.0. To align with the factorial experimental design, endpoint outcomes were analyzed using a two‐way ANOVA with Diet (ND vs. WD) and Exposure (Control vs. PM_2.5_) as the main factors. The interaction term (Diet × Exposure) was explicitly tested. Following significant main effects or interactions, Tukey's multiple comparisons test was performed to evaluate differences between individual groups. For graphical presentation, significant differences between groups are indicated by distinct lower‐case letters (e.g., ‘a’ vs. ‘b’); groups sharing the same letter are not significantly different (*p* > 0.05). For time‐dependent variables (body weight, IPGTT), mixed‐effects models were employed. For categorical data, specifically the incidence of positive proteinuria, statistical comparisons between groups were performed using Fisher's exact test. A *p*‐value < 0.05 was considered statistically significant.

## 3 Results

### 3.1 PM_2.5_ Does Not Affect Body Weight Trajectories and Tissue Weights Induced by a Western Diet in Mice

After 7 months of WD induction combined with thrice‐weekly PM_2.5_ exposure, mice in the WD and WD + PM groups exhibited significant increases in body weight growth, liver weight, and epididymal white adipose tissue (eWAT) compared to the control (ND) group (Figure 1A–C). However, PM_2.5_ exposure alone did not result in significant differences in body weight trajectories, liver weight, or adipose tissue weight compared to the ND group. Moreover, the co‐exposure to PM_2.5_ in WD‐fed mice did not further increase these parameters throughout the experimental period. Kidney weight remained consistent across all experimental groups (Figure 1D). We further assessed the macroscopic phenotype of the liver through gross morphological examination (Figure 1E). Livers from the ND and ND + PM groups exhibited a normal dark‐red appearance and smooth texture. In contrast, livers from the WD and WD + PM groups were visibly enlarged and displayed a distinct pale, yellowish discoloration characteristic of severe steatosis. This macroscopic phenotype visually confirms the quantitative increase in liver weight and supports the histological findings of lipid accumulation. Regarding the kidneys, gross inspection revealed no remarkable differences across the groups (Figure 1F). All kidneys retained a consistent reddish‐brown color and smooth surface, without overt signs of atrophy, cysts, or discoloration. These macroscopic observations align with the unchanged kidney weights, indicating that the renal alterations in this model do not manifest as gross structural deformities.

**FIGURE 1:**
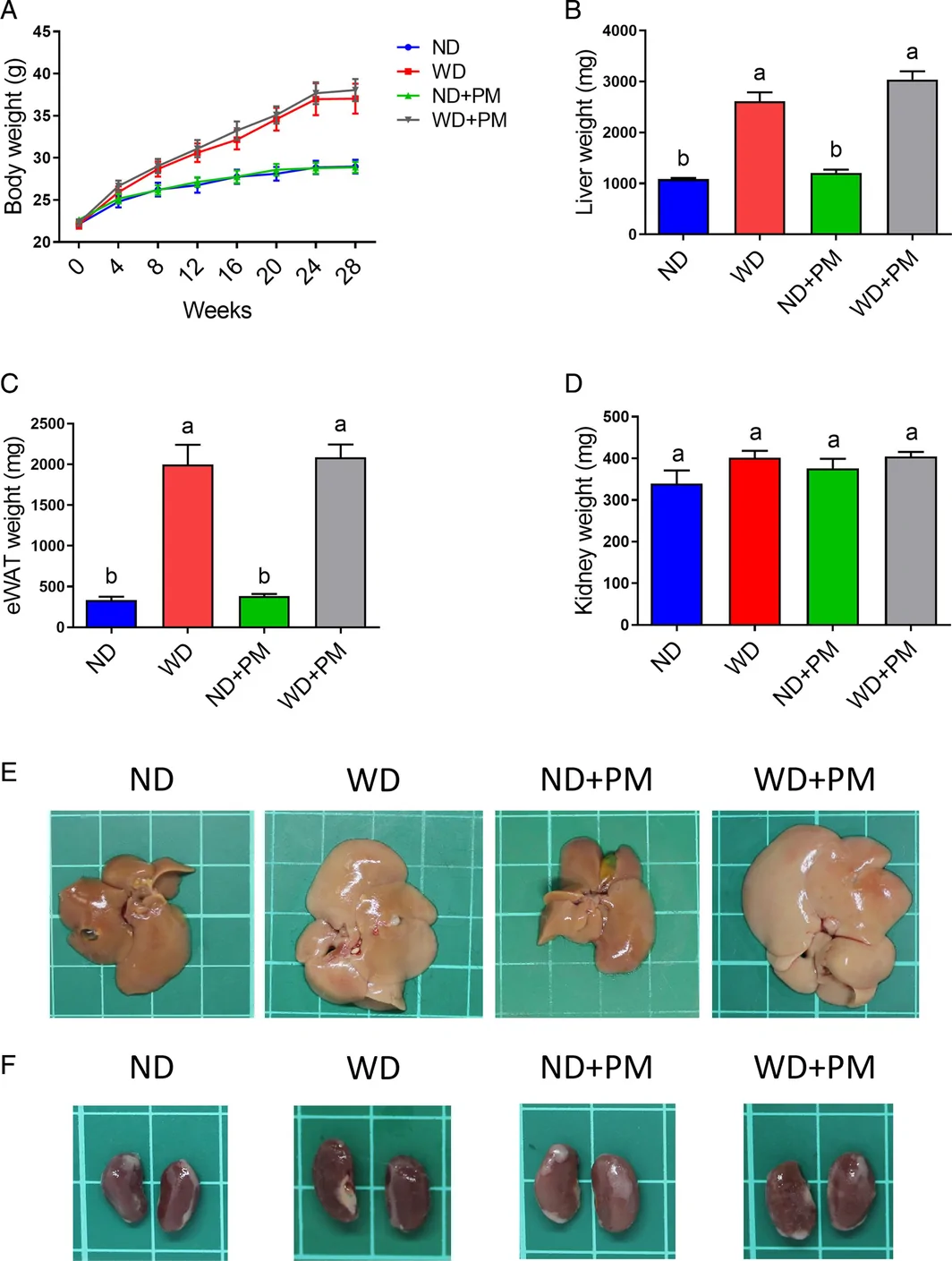
Impact of western diet and PM_2.5_ exposure on body and organ weights in mice. (A) Body weight, (B) liver weight, (C) eWAT weight, and (D) kidney weight were measured after 7 months of dietary intervention and thrice‐weekly PM_2.5_ exposure. (E) Representative gross morphological photographs of livers and (F) kidneys from each experimental group (scale: Background grid = 1 cm). Data are presented as mean ± SEM. Statistical analysis for body weight trajectories (A) was performed using a mixed‐effects model, while endpoint organ weights (B–D) were analyzed using two‐way ANOVA. In all panels, differences between individual groups were determined by Tukey's multiple comparisons test. Different letters indicate significant differences (*p* < 0.05); groups sharing the same letter are not significantly different. Two‐way ANOVA revealed significant main effects for Diet on body, liver, and eWAT weights, but no significant Diet × PM_2.5_ interaction was observed (*p* > 0.05).

### 3.2 Combined Effects of PM_2.5_ Exposure and Western Diet on Glucose Regulation

Fasting blood glucose was significantly elevated by WD compared with ND controls, whereas PM_2.5_ exposure alone did not alter fasting glucose. Importantly, the WD + PM group did not differ from WD alone, indicating no additive effect of PM_2.5_ on WD‐induced hyperglycemia (Figure 2A). Fasting insulin was robustly increased in WD‐fed mice; PM_2.5_ alone had no effect, and insulin levels in WD + PM mice remained elevated versus ND but did not differ from WD (Figure 2B). A similar pattern was seen for HOMA‐IR: WD feeding induced a marked rise in insulin resistance; PM_2.5_ alone had no significant impact, and the WD + PM group was not significantly different from WD (Figure 2C). To comprehensively evaluate the impact of PM_2.5_ on systemic glucose homeostasis, we performed an Intraperitoneal Glucose Tolerance Test (IPGTT) and calculated the corresponding Area Under the Curve (AUC). Analysis of the time‐dependent glucose profiles using a mixed‐effects model, alongside two‐way ANOVA for the AUC, yielded consistent findings. As expected, mice fed a Western diet exhibited significant glucose intolerance, characterized by sustained hyperglycemia during the tolerance test and a marked increase in AUC compared to normal diet controls. However, chronic PM_2.5_ exposure did not further exacerbate this metabolic deficit. The glucose excursion curves of the WD + PM group were indistinguishable from those of the WD group at all time points measured. Furthermore, quantitative analysis of the AUC confirmed that PM_2.5_ co‐exposure failed to induce any additional deterioration in overall glucose handling in Western diet‐fed mice (Figure 2D,E).

**FIGURE 2:**
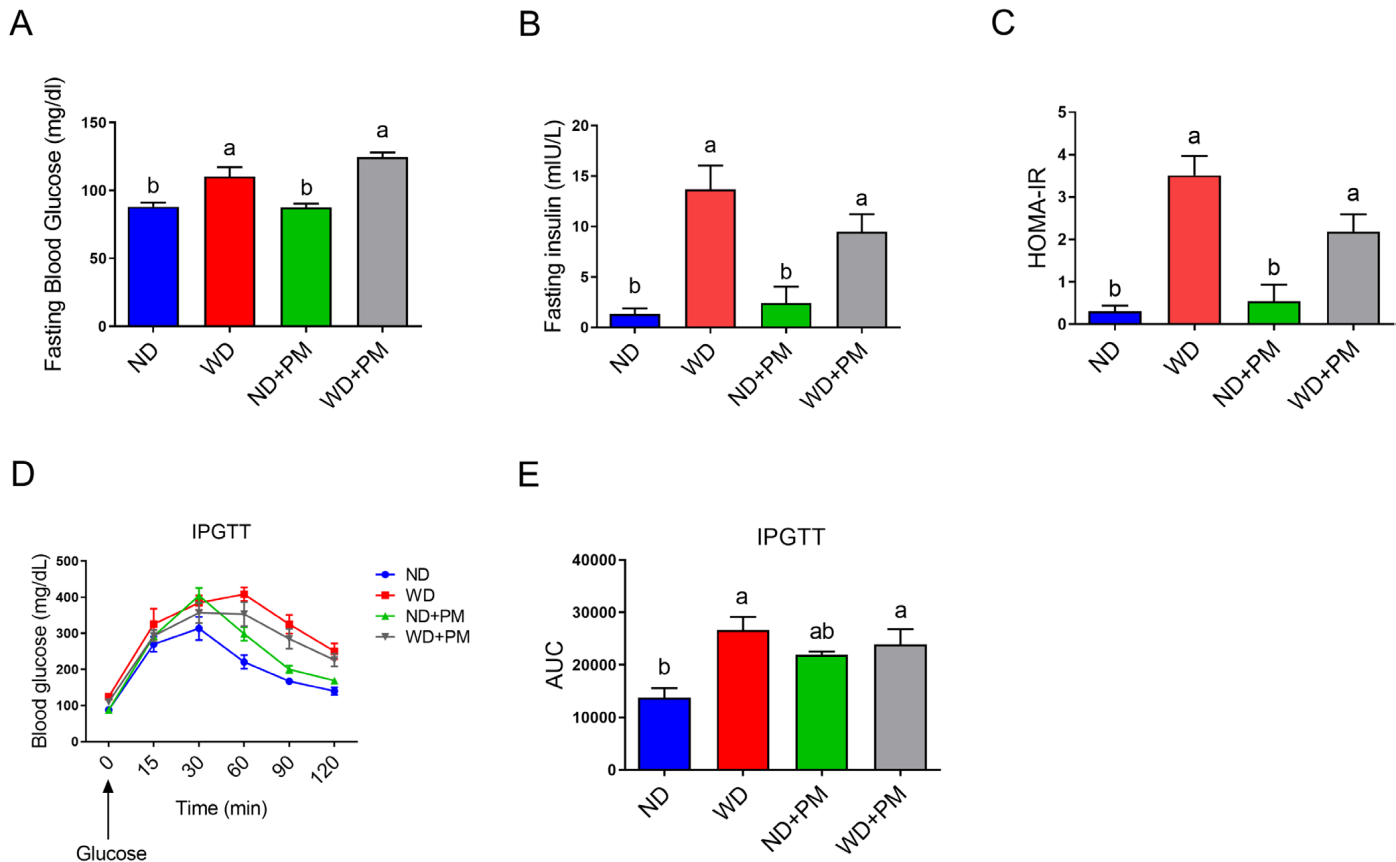
Effects of western diet and PM_2.5_ exposure on glucose metabolism in mice. (A) Fasting blood glucose, (B) fasting insulin levels, (C) HOMA‐IR (Homeostatic Model Assessment of Insulin Resistance), (D) intraperitoneal glucose tolerance test (IPGTT), and (E) area under the curve (AUC) quantification of IPGTT were measured in mice after 7 months of dietary intervention (ND or WD) with or without thrice‐weekly PM_2.5_ exposure. IPGTT was conducted to assess glucose tolerance, and AUC was calculated to quantify overall glucose excursion. Data are presented as mean ± SEM. Statistical analysis for the IPGTT glucose excursion curve (D) was performed using a mixed‐effects model, while endpoint metabolic parameters (A–C, E) were analyzed using standard two‐way ANOVA. In all panels, differences between individual groups were determined by Tukey's multiple comparisons test. Different letters indicate significant differences (*p* < 0.05); groups sharing the same letter are not significantly different. Regarding the interaction analysis, two‐way ANOVA indicated no significant Diet × PM_2.5_ interaction for any of the assessed glucose metabolic parameters (*p* > 0.05).

### 3.3 Western Diet Alters Serum Lipid Profiles, With PM_2.5_ Co‐Exposure Modestly Enhancing HDL‐C Levels

To assess the impact of chronic PM_2.5_ exposure on WD‐induced alterations in lipid metabolism, we measured serum biochemical markers including triglycerides (TG), total cholesterol (TCHO), and high‐density lipoprotein cholesterol (HDL‐C). Triglyceride levels showed no apparent differences among the groups (Figure 3A). Total cholesterol levels were significantly elevated in the WD and WD + PM groups compared to the ND and ND + PM groups. While total cholesterol levels in the WD + PM group exhibited a slight increase relative to the WD group, this difference did not reach statistical significance (Figure 3B). In contrast, a distinct interaction pattern was observed for HDL‐C. Analysis by two‐way ANOVA revealed a highly significant Diet × PM_2.5_ interaction (*p* < 0.001). While PM_2.5_ exposure in ND‐fed mice had no impact on HDL‐C levels compared to controls, co‐exposure in WD‐fed mice resulted in a marked and statistically significant further elevation compared to the WD group alone (*p* < 0.0001). These results indicate that while the Western diet is the primary driver of hypercholesterolemia, PM_2.5_ acts synergistically with the dietary background to specifically exacerbate the elevation of HDL‐C (Figure 3C).

**FIGURE 3:**
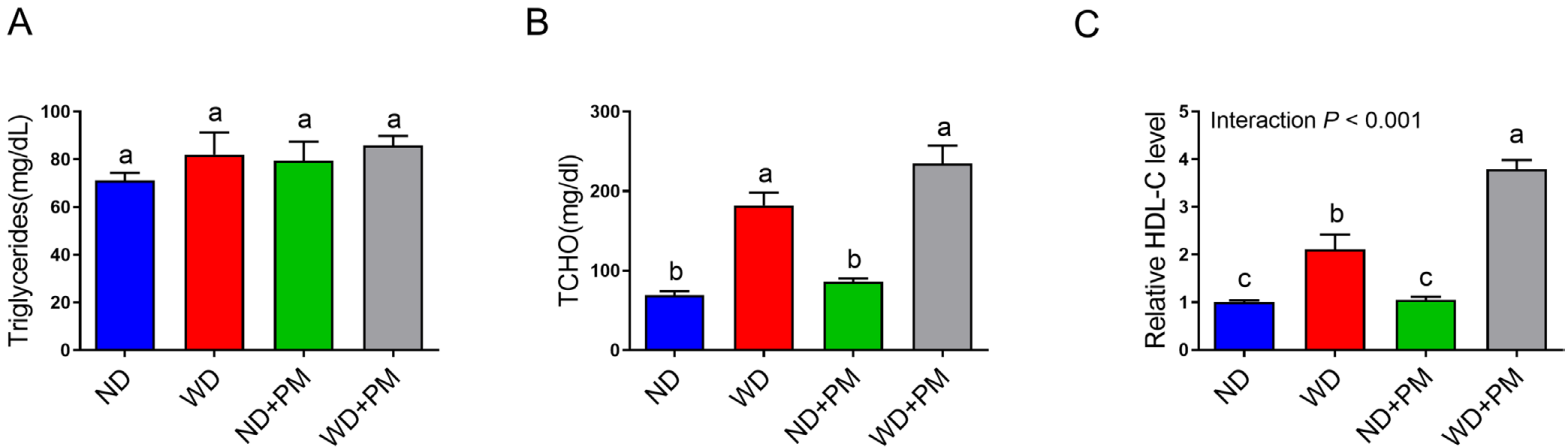
Changes in serum lipid parameters following western diet and PM_2.5_ exposure. Serum levels of (A) triglycerides (TG), (B) total cholesterol (TCHO), and (C) high‐density lipoprotein cholesterol (HDL‐C) were measured in mice after 7 months of dietary intervention (ND or WD) with or without thrice‐weekly PM_2.5_ exposure. Data are presented as mean ± SEM. Statistical significance was determined by two‐way ANOVA followed by Tukey's post hoc test. Different letters indicate significant differences between groups (*p* < 0.05); groups sharing the same letter are not significantly different. Two‐way ANOVA revealed a highly significant Diet × PM_2.5_ interaction for HDL‐C levels (*p* < 0.001), whereas no significant interaction was observed for TG or TCHO.

### 3.4 Aggravated Hepatic Inflammatory Responses Induced by Chronic PM_2.5_ and Western Diet Co‐Exposure

Hematoxylin and eosin (H&E) staining (Figure 4A) revealed distinct hepatic histological differences among the experimental groups. To rigorously assess the extent of pathology, quantitative image analysis was performed for both steatosis and inflammatory infiltration. In terms of lipid accumulation, the ND and ND + PM groups showed minimal steatosis. The WD group exhibited severe macrovesicular steatosis, characterized by large lipid droplets displacing hepatocyte nuclei. Quantitative analysis confirmed that this steatotic phenotype was driven exclusively by the dietary factor. The WD and WD + PM groups exhibited equivalent degrees of steatosis (~22% area), with no statistical difference observed between them (Figure 4B). This indicates that while PM_2.5_ exposure promotes other pathological changes, it does not further increase the volume of lipid accumulation induced by the Western diet. In contrast, quantitative analysis of immune cell infiltration revealed a distinct pattern of exacerbation (Figure 4C). PM_2.5_ exposure alone (ND + PM) significantly increased the area of immune cell infiltration compared to the ND control (*p* < 0.05), establishing PM_2.5_ as an independent pro‐inflammatory factor. Notably, the WD + PM group exhibited the highest degree of immune cell infiltration among all groups. Although the statistical interaction between diet and PM_2.5_ showed a trend (*p* = 0.0886) rather than strict significance—suggesting an additive rather than synergistic mechanism—post hoc analysis confirmed a highly significant elevation in infiltration area in the WD + PM group compared to the WD group (*p* < 0.0001). This demonstrates that PM_2.5_ exposure superimposes a significant inflammatory burden on top of diet‐induced steatosis, leading to a more severe overall hepatic phenotype. This histological observation was supported by a further elevation in serum alanine aminotransferase (ALT) levels in the WD + PM group compared to the WD group (Figure 4D), indicating increased hepatocellular injury. To complement the histological and biochemical evidence of hepatic inflammation, we analyzed the expression patterns of representative inflammatory cytokines at the transcriptional level. Consistently, the expression of TNF‐α, IL‐1β, and CCL2 mRNA levels (Figure 4E–G) were significantly upregulated in both the WD and WD + PM groups relative to the ND and ND + PM groups. However, the response patterns differed among specific cytokines. Analysis by two‐way ANOVA revealed a significant Diet × PM_2.5_ interaction for both TNF‐α and CCL2 (*p* < 0.0001). Unlike the additive pattern observed in IL‐1β—where expression was driven primarily by diet and did not differ significantly between WD and WD + PM groups—TNF‐α and CCL2 exhibited a distinct synergistic characteristic. The WD + PM group displayed a dramatic surge in the expression of these two markers, which was significantly higher than in the WD group. This suggests that while the Western diet provides the baseline inflammatory stress, PM_2.5_ co‐exposure acts synergistically to specifically potentiate the TNF‐α and CCL2 signaling cascades.

**FIGURE 4:**
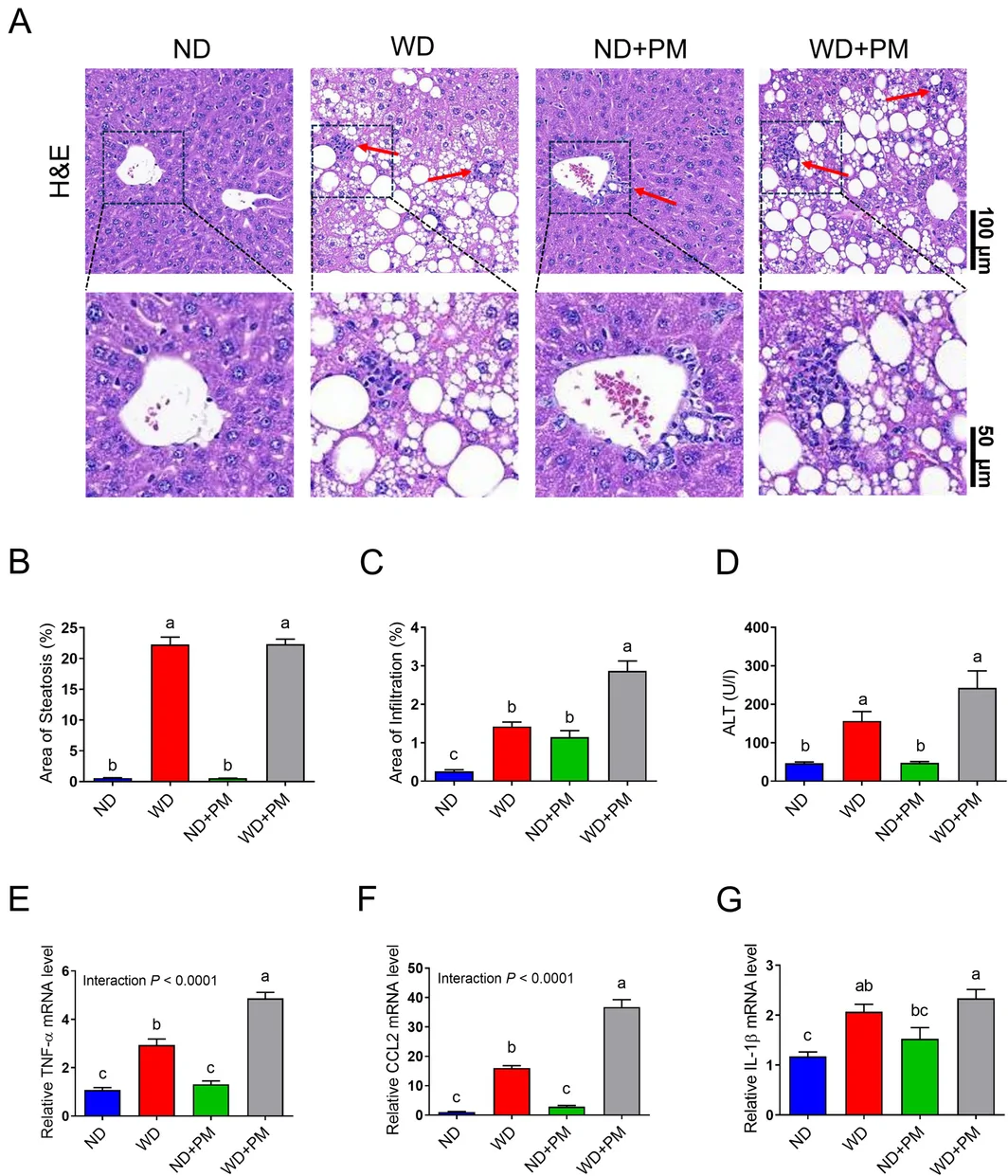
PM_2.5_ co‐exposure enhances hepatic inflammation in mice fed a western diet. (A) Hematoxylin and eosin (HE) staining of liver sections showing hepatic morphology across experimental groups; red arrows indicate areas of immune cell infiltration. (B) Quantification of the percentage area of hepatic steatosis. (C) Quantification of the percentage area of immune cell infiltration. (D) Serum alanine aminotransferase (ALT) levels measured at the endpoint as a marker of hepatocellular injury. (E) TNF‐α, (F) CCL2, and (G) IL‐1β mRNA expression levels were measured in liver tissue to assess hepatic inflammatory responses in mice following 7 months of dietary intervention (ND or WD), with or without thrice‐weekly PM_2.5_ exposure. Data are presented as mean ± SEM. Statistical significance was determined by two‐way ANOVA followed by Tukey's post hoc test. Different letters indicate significant differences between groups (*p* < 0.05); groups sharing the same letter are not significantly different. Two‐way ANOVA demonstrated a significant Diet × PM_2.5_ interaction for the mRNA expression of TNF‐α and CCL2 (*p* < 0.0001). No significant interaction was detected for hepatic steatosis, immune cell infiltration, ALT or IL‐1β levels (*p* > 0.05).

### 3.5 Chronic PM_2.5_ Exposure Enhances Western Diet‐Induced Hepatic Fibrosis

Masson's trichrome staining (Figure 5A) showed minimal collagen deposition in the ND and ND + PM groups. The WD group exhibited mild collagen accumulation in pericentral and limited perisinusoidal areas, but quantitative analysis (Figure 5B) revealed no statistically significant increase. In contrast, the WD + PM group demonstrated a significant elevation in collagen deposition compared to all other groups. To further elucidate the molecular mechanisms underlying the enhanced fibrotic response, we examined the expression of key fibrogenic genes. The expression levels of TGF‐β, α‐SMA, and COL1A1 (Figure 5C–E) were significantly upregulated in the WD‐fed groups; however, their regulatory patterns exhibited distinct differences. For the upstream profibrotic cytokine TGF‐β, analysis by two‐way ANOVA revealed significant main effects for both diet and PM_2.5_ (*p* < 0.0001), with no statistically significant interaction (*p* = 0.16). While this lack of interaction suggests an overall additive pattern, post hoc analysis indicated that the impact of PM_2.5_ was context‐dependent: the elevation in TGF‐β expression did not reach statistical significance in the ND background (*p* = 0.0798) but was highly significant in the WD background (*p* < 0.001). Consequently, the WD + PM group exhibited the highest TGF‐β levels, significantly exceeding those of the WD group, reflecting the cumulative burden of both stressors. In contrast, the downstream markers of hepatic stellate cell activation (α‐SMA) and collagen synthesis (COL1A1) exhibited a significant Diet × PM_2.5_ interaction (*p* < 0.05). The WD + PM group showed a synergistic upregulation of these executive markers that was disproportionately higher than in the WD group. This suggests that while TGF‐β signaling is amplified in an additive manner, PM_2.5_ co‐exposure acts synergistically to potentiate the downstream cascade of myofibroblast activation and matrix deposition. To explore potential differences between fibrotic gene expression and histological collagen accumulation, we further analyzed ECM remodeling genes, MMP13 and TIMP1 (Figure 5F,G). Both MMP13, a collagen‐degrading enzyme, and TIMP1, an inhibitor of matrix metalloproteinases, were significantly upregulated in the WD and WD + PM groups. Notably, MMP13 expression was relatively higher in the WD group, while TIMP1 expression was more pronounced in the WD + PM group. This reciprocal pattern may reflect the enhanced collagen deposition observed histologically in the WD + PM group.

**FIGURE 5:**
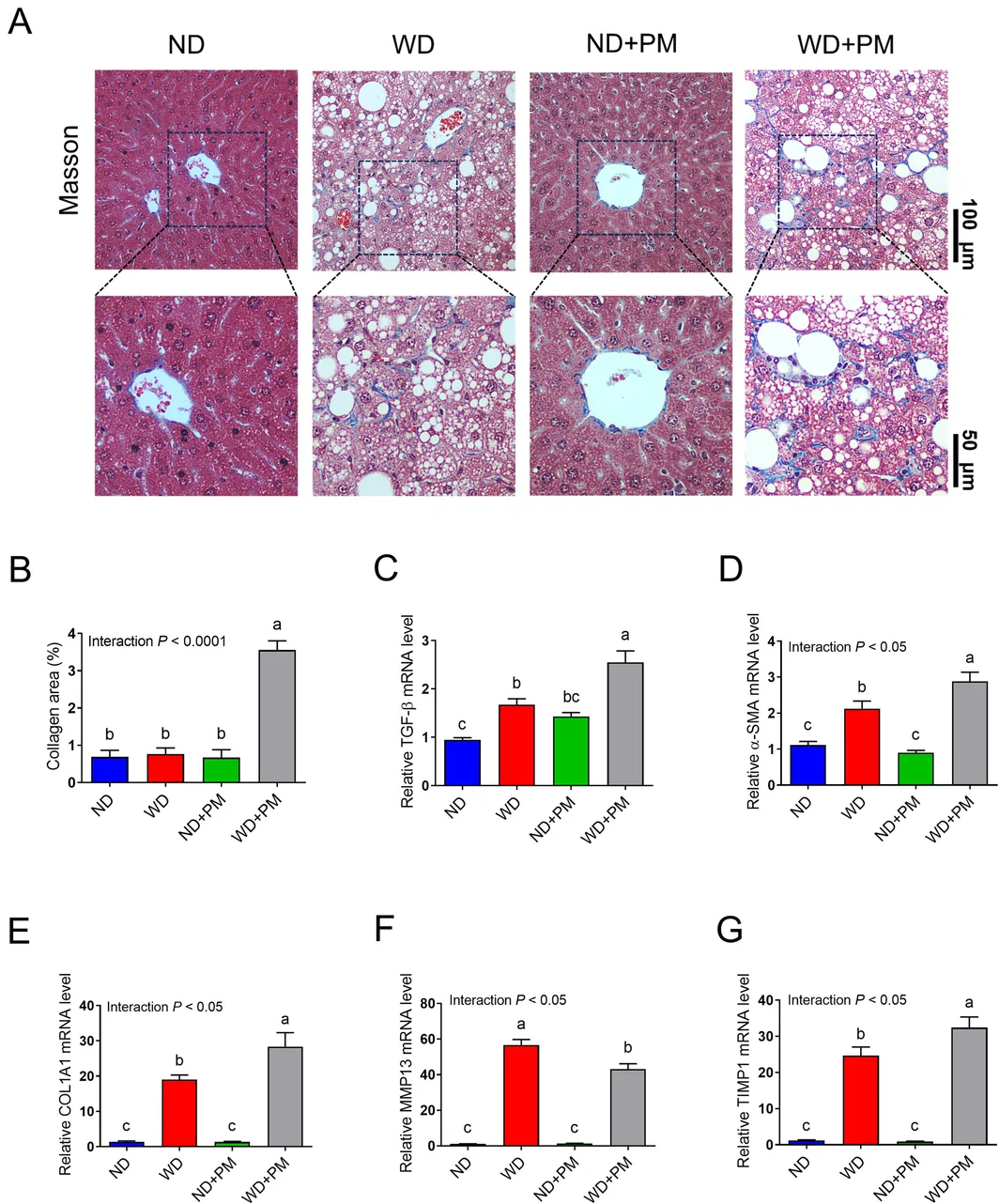
PM_2.5_ co‐exposure potentiates hepatic fibrosis in mice fed a western diet. (A) Masson's trichrome staining illustrating collagen deposition and fibrosis. (B) Quantification of collagen‐positive area from Masson's trichrome‐stained sections, presented as percentage of total tissue area. (C) TGF‐β, (D) α‐SMA, (E) COL1A1, (F) MMP13, (G) TIMP1 mRNA expression levels were measured in liver tissue to assess hepatic fibrogenic gene expression in mice following 7 months of dietary intervention (ND or WD), with or without thrice‐weekly PM_2.5_ exposure. Data are presented as mean ± SEM. Statistical significance was determined by two‐way ANOVA followed by Tukey's post hoc test. Different letters indicate significant differences between groups (*p* < 0.05); groups sharing the same letter are not significantly different. Two‐way ANOVA revealed a significant Diet × PM_2.5_ interaction for α‐SMA, COL1A1, MMP13, and TIMP1 expression (*p* < 0.05), indicating a synergistic effect on hepatic fibrotic remodeling. In contrast, TGF‐β expression showed significant main effects for both factors but no significant interaction (*p* = 0.16).

### 3.6 Effects of PM_2.5_ and Western Diet on Kidney Function in Mice

Renal function is influenced by both dietary and environmental factors, with WD and PM_2.5_ exposure each associated with metabolic and renal impairments. To assess their combined effects, we evaluated serum and urinary renal biomarkers and performed histopathological analysis. Hematoxylin and eosin (H&E) staining (Figure 6A) showed normal renal architecture in the ND and ND + PM groups, with intact glomeruli and tubules and no notable abnormalities. In contrast, the WD group exhibited vacuolation predominantly in the interstitial regions, a change also observed in the WD + PM group. No significant immune cell infiltration or additional morphological changes were seen in the PM_2.5_‐exposed groups, suggesting that PM_2.5_ alone does not induce or exacerbate structural kidney damage. Given the presence of interstitial vacuolation, we next examined urinary kidney injury molecule‐1 (KIM‐1), a sensitive marker of early tubular damage. KIM‐1 levels were elevated in both WD and WD + PM groups compared to controls, with the WD + PM group showing the highest levels among all groups. However, this increase in the WD + PM group did not reach statistical significance compared to WD alone (Figure 6B). To further explore potential functional consequences, we next analyzed serum creatinine levels as an indicator of renal filtration function. After 7 months of exposure, creatinine concentrations were significantly higher in the ND + PM and WD + PM groups compared to their respective controls (Figure 6C), with no significant difference between the two PM_2.5_‐exposed groups. In addition to serum creatinine, urinary microalbumin and creatinine concentrations were quantified to assess glomerular function, and used to calculate the urine albumin‐to‐creatinine ratio (uACR). Urinary microalbumin levels were slightly higher in the WD and WD + PM groups, with the highest levels observed in WD + PM, though differences among groups were minimal (Figure 6D). Urinary creatinine levels were also mildly elevated in WD + PM (Figure 6E), and these combined changes contributed to the highest uACR values in WD + PM, followed by WD, while overall group differences remained small (Figure 6F). Proteinuria was not detected in the ND group by urine dipstick screening. In contrast, proteinuria was observed in 28.6% of WD mice, 57.1% of ND + PM mice, and 75% of WD + PM mice. Fisher's exact test revealed a significant difference among groups (*p* = 0.014; Table 1), suggesting a potential association between chronic PM_2.5_ exposure and increased urinary protein excretion, particularly under the stress of a Western diet.

**FIGURE 6:**
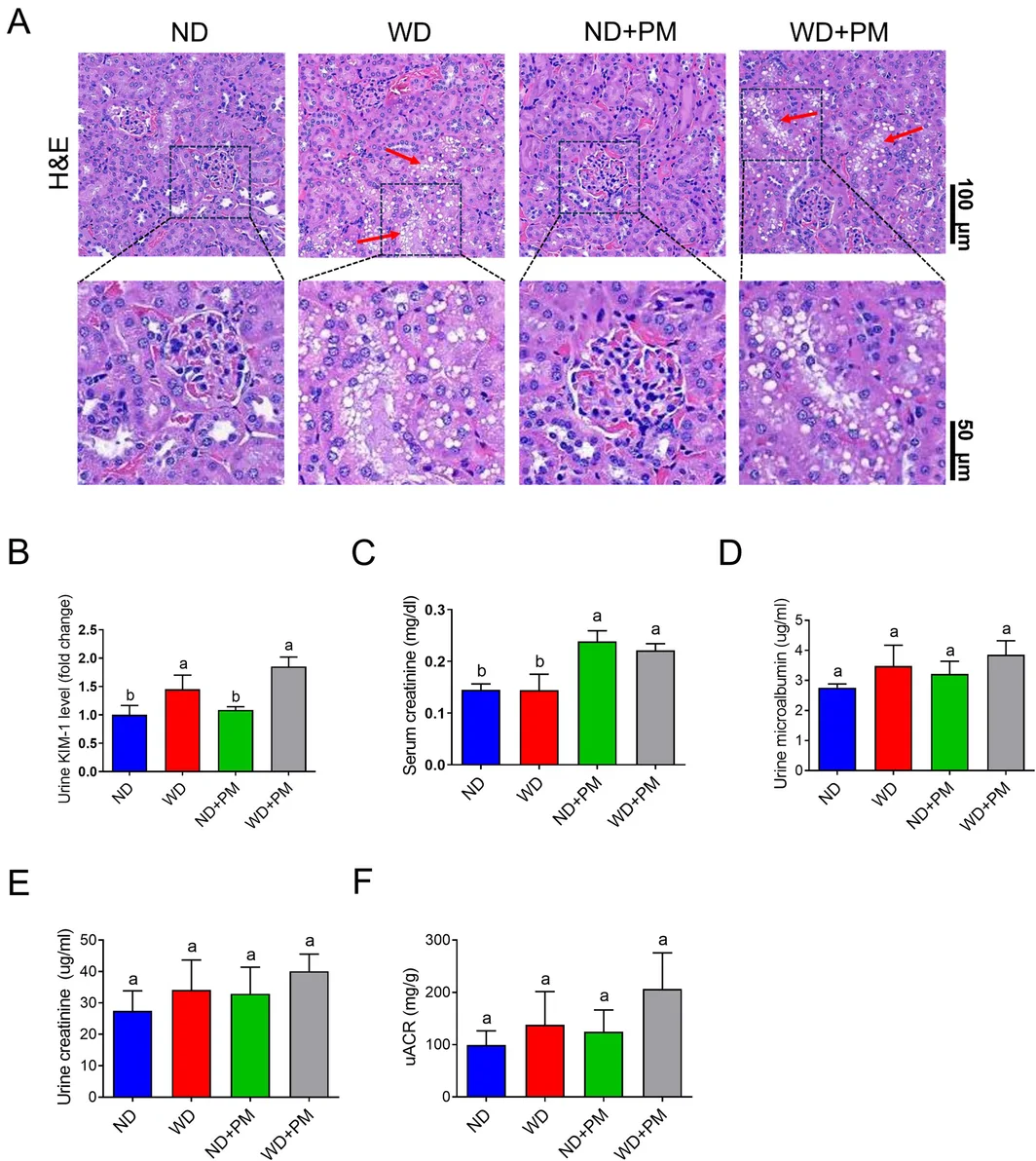
Effects of a western diet and PM_2.5_ exposure on renal histopathology and biochemical markers in mice. (A) Representative hematoxylin and eosin (H&E) staining of mouse kidney tissues from different groups; red arrows indicate interstitial vacuolation. (B) Urinary KIM‐1, (C) serum creatinine, (D) urinary microalbumin, (E) urinary creatinine, and (F) urine albumin‐to‐creatinine ratio (uACR) were analyzed to assess renal function in mice after 7 months of dietary intervention (ND or WD) with or without thrice‐weekly PM_2.5_ exposure. Data are presented as mean ± SEM. Statistical significance was determined by two‐way ANOVA followed by Tukey's post hoc test. Different letters indicate significant differences between groups (*p* < 0.05); groups sharing the same letter are not significantly different. Two‐way ANOVA indicated no significant Diet × PM_2.5_ interaction for the assessed renal histological and biochemical markers (*p* > 0.05), suggesting that dietary and environmental factors exert largely independent effects on the kidney in this model.

**TABLE 1:** Proteinuria incidence in mice after western diet and PM_2.5_ exposure.

Group	Negative (*n*, %)	Positive (*n*, %)	Exact sig. (2 sided)
ND	7 (100%)	0 (0%)	
WD	5 (71.4%)	2 (28.6%)	
ND + PM	3 (42.9%)	4 (57.1%)	
WD + PM	2 (25.0%)	6 (75.0%)	
Fisher exact test			0.014

*Note:* Data are presented as number (percentage). Statistical significance was determined by Fisher's exact test. Sample sizes for urinary analysis were *n* = 7–8 per group; specific samples were excluded from the analysis due to visible fecal contamination during the 24‐h collection period.

## 4 Discussion

Our findings suggest that chronic, moderate‐level exposure to PM_2.5_—at levels designed to mimic real‐world human scenarios—exacerbates WD‐induced MASLD and independently contributes to early‐stage renal dysfunction. In dissecting these effects, our study identifies the WD as the primary driver of systemic metabolic pathology, including glucose intolerance and insulin resistance, with PM_2.5_ co‐exposure showing no additive impact on these glycemic parameters. However, for hepatic pathology, PM_2.5_ acts as a distinct modifier that compounds the dietary effects, leading particularly to exacerbated hepatic inflammation and fibrosis. In the kidney, PM_2.5_ exposure appears to drive a functional decline distinct from the diet‐driven structural changes. This exacerbating role is underscored by our observation that prolonged PM_2.5_ exposure in the absence of a WD did not induce significant fibrosis, indicating that environmental pollutants are crucial aggravating factors for pre‐existing metabolic conditions. This demonstration is particularly significant as our study's use of prolonged, environmentally relevant exposure levels is a key distinction from the existing literature, which has predominantly focused on short‐term exposures to supraphysiological concentrations. Therefore, our approach offers critical new insights into how sustained, real‐world air pollution can subtly influence metabolic processes and predispose individuals to more severe health complications over time.

One of the more paradoxical findings in our study was the unexpected increase in HDL‐C levels in both the WD and WD + PM groups. Interestingly, similar trends have been reported in various animal studies where high‐fat or high‐fat/high‐cholesterol diets, like the Western diet, increased circulating HDL‐C levels [25, 26, 27]. This elevation is thought to reflect an adaptive metabolic response to dietary lipid overload. One proposed mechanism involves the upregulation of apolipoprotein A‐I (apoA‐I) production and stabilization, which enhances HDL particle formation and reduces its clearance [26]. This is supported by experimental models, which have shown that high‐fat feeding can enhance HDL cholesterol ester transport and reduce apoA‐I catabolism, resulting in increased plasma HDL‐C levels. In addition, high‐fat/high‐cholesterol diets can induce hepatic free cholesterol accumulation, which may stimulate the formation of apoE‐rich HDL particles [27]. These compositional and kinetic changes in HDL may support enhanced reverse cholesterol transport, although the functional integrity of such HDL remains uncertain under conditions of metabolic stress. In our study, while the HDL‐C elevation was also observed in the WD + PM group, it was even more pronounced. This finding aligns with some human studies reporting elevated HDL‐C after short‐ to medium‐term PM_2.5_ exposure in certain populations, though these changes are not consistently observed, with other studies reporting reductions or no significant change [28, 29, 30].

However, this elevation may represent dysfunctional HDL, as PM_2.5_ and metabolic stress are known to impair HDL's protective function [31, 32]. This dysfunction is more clinically relevant than HDL‐C levels alone [33] and may be driven by the suppression of key antioxidant enzymes like paraoxonase‐1 (PON1) [34, 35]. Thus, the HDL‐C increase likely masks a detrimental loss of function, warranting further investigation into its functionality.

Our study reveals a multi‐layered renal pathology where PM_2.5_ induced early‐stage dysfunction (elevated creatinine) even without major histological alterations, while WD induced structural vacuolation. Interestingly, these histological alterations in the WD + PM group were similar to those observed in the WD group, suggesting that the Western diet primarily drives these early structural changes. Although urinary biomarkers (KIM‐1, uACR) showed a trend towards elevation in the WD + PM group, these differences did not reach statistical significance compared to the WD group, aligning with the histological observation that structural damage (vacuolation) was primarily diet‐driven. Nevertheless, the significant rise in serum creatinine and the increased frequency of overt proteinuria in the WD + PM group point to a ‘functional nephrotoxicity.’ This suggests that environmentally relevant pollution may compromise renal filtration capacity and glomerular integrity, potentially prior to the onset of massive structural collapse or profound biomarker elevation. Thus, PM_2.5_ acts as an independent stressor that superimposes a functional burden on top of the metabolic‐driven structural injury. Therefore, the key insight from our study is not merely that biomarkers are important, but rather that environmentally relevant pollution can initiate a sub‐pathological ‘functional nephrotoxicity,’ experimentally demonstrating that functional decline can precede overt structural kidney damage [36]. Furthermore, the addition of metabolic dysfunction can precipitate a shift from a purely functional injury to a combined functional‐structural pathology, providing a tangible in vivo model of this progression.

Our results align with growing public health concerns regarding the impact of long‐term, moderate PM_2.5_ exposure. Translating our mouse model findings into human‐equivalent exposure levels suggests that prolonged exposure to PM_2.5_ concentrations commonly encountered in regions where air quality is classified as ‘Unhealthy for Sensitive Groups’ (AQI ~135)—reflecting persistent moderate pollution—may be sufficient to exacerbate diet‐induced metabolic dysfunction while independently contributing to renal changes over time. Although each exposure event may seem minor, the cumulative effect poses significant health risks. The public health implications of this research are both direct and significant. Our study provides crucial experimental evidence that individuals with pre‐existing metabolic dysfunction are more vulnerable to the adverse effects of moderate air pollution, experiencing accelerated progression of both liver and kidney disease. Therefore, a key policy recommendation emerging from our work is that national and international air quality guidelines should consider expanding the formal definition of ‘sensitive groups’ to explicitly include individuals with metabolic syndrome, MASLD, or early‐stage CKD. This classification, which already includes patients with cardiovascular and pulmonary diseases, would ensure that public health alerts and exposure‐reduction advisories are more accurately targeted to this large and growing at‐risk population, thus providing a more effective strategy to mitigate their long‐term health burden from environmental pollution.

Despite the valuable insights provided, this study has several specific limitations that warrant discussion. First, our use of a standardized PM_2.5_ source (NIST SRM 1650b) does not fully replicate the complex and regionally variable composition of real‐world ambient PM_2.5_. However, this specific reference material was intentionally chosen primarily for its well‐characterized particle size distribution. With a mass median diameter of approximately 0.18 μm, SRM 1650b is highly relevant for PM_2.5_‐related research, ensuring that the exposure material consists predominantly of fine particles capable of penetrating deep into the lower respiratory tract. Second, the administration via tracheal instillation, while ensuring accurate dosing, delivers a bolus dose that differs from the physiological kinetics of real‐world inhalation. Nonetheless, for a more authentic exposure scenario, future studies should employ concentrated ambient particles and whole‐body inhalation systems. Finally, this study was conducted exclusively in male mice. Given the known sex‐specific differences in both metabolic diseases and physiological responses to pollutants [37, 38, 39], our findings may not be generalizable to females, highlighting the need for future investigations to include both sexes.

In conclusion, our study demonstrates that under a chronic, moderate‐level exposure paradigm, PM_2.5_ acts as a distinct hepatic stressor, specifically exacerbating the inflammatory and fibrotic progression of diet‐induced MASLD without further worsening systemic insulin resistance, while independently contributing to early‐stage renal dysfunction. While acknowledging the inherent limitations of translating animal findings to humans, the primary contribution of this work lies in utilizing an exposure model with a dose and duration that are more representative of chronic human exposure scenarios than those often used in previous animal studies. The value of this approach is in providing a potential platform for further investigation. The subtle, yet significant, hepato‐renal changes observed in our model suggest that biological samples generated through this paradigm could be a valuable resource for future, exploratory omics‐based analyses. Such studies may help identify potential biomarkers or pathways relevant to the early stages of disease progression in individuals facing long‐term metabolic and environmental challenges.

## Funding

This work was supported by research grants KMU‐TB114005, NK114P30, NSYSU‐KMU‐115‐P10 and KMU‐TB114005‐2 from Kaohsiung Medical University, and by the National Science and Technology Council (NSTC), Taiwan (grant number NSTC 113‐2320‐B‐037‐018 and NSTC 114‐2314‐B‐037‐092), and was partly supported by the “Center of Excellence for Metabolic Associated Fatty Liver Disease, National Sun Yat‐sen University, Kaohsiung” from The Featured Areas Research Center Program within the framework of the Higher Education Sprout Project by the Ministry of Education (MOE) in Taiwan.

## Conflicts of Interest

The authors declare no conflicts of interest.

## Data Availability

The data that support the findings of this study are available from the corresponding author upon reasonable request.
